# The cost of dengue shock and septic shock in Vietnam: a patient-centred economic analysis

**DOI:** 10.1093/inthealth/ihaf105

**Published:** 2026-05-08

**Authors:** Angela McBride, Nguyen Van Hao, Phan Vinh Tho, Luong Thi Hue Tai, Nguyen Thanh Phong, Nguyen Thanh Ngoc, Nguyen Anh Huyen, Trinh Manh Hung, Martin J. Llewelyn, Sophie Yacoub, Louise Thwaites, Hugo C. Turner

**Affiliations:** a Department of Global Health and Infection, Brighton and Sussex Medical School, Brighton, UK, BN1 9PX; b Dengue and Emerging Infection groups, Oxford University Clinical Research Unit, Ho Chi Minh City, Vietnam, 700000; c Center for Tropical Medicine and Global Health, Nuffield Department of Medicine, University of Oxford, Oxford, UK, OX3 7LG; d Hospital for Tropical Diseases, Ho Chi Minh City, Vietnam, 700000; e University of Medicine and Pharmacy at Ho Chi Minh City, Ho Chi Minh City, Vietnam, 700000; f Queensland Alliance for Environmental Health Sciences, University of Queensland, Brisbane, Australia, QLD 4102; g MRC Centre for Global Infectious Disease Analysis, School of Public Health, Imperial College London, London, UK, W12 0BZ

**Keywords:** catastrophic costs, catastrophic health expenditure, economic, septic shock, severe dengue, Vietnam

## Abstract

**Background:**

Dengue shock (DS) and septic shock (SS) are the most common infectious causes of shock in Vietnam. Little is known about the cost of an episode of DS or SS from the patient perspective. We aimed to describe the direct medical, non-medical and productivity costs associated with DS and SS.

**Methods:**

We recruited adults with DS and SS to a prospective observational study at the Hospital for Tropical Diseases, Ho Chi Minh City, from 2019 to 2021. We collected hospital bills, insurance status and out of pocket payments, and conducted an economic questionnaire at discharge, 1, 3 and 6 mo later. We calculated the proportion incurring catastrophic health expenditure (CHE) and catastrophic costs.

**Results:**

We recruited 127 adults with DS, of whom 118 survived, and 35 with SS, of whom 24 survived; 18.9% and 71.4% with DS and SS, respectively, incurred CHE. When non-medical and productivity costs were considered, the true cost of illness was 6.3 and 6.7 times higher than the hospital bill for DS and SS, respectively.

**Conclusions:**

Productivity costs must be counted when assessing the cost of critical illness in low- and lower-middle income countries. It is vital that financial protection systems are extended to cover patients requiring high-cost critical care in Vietnam.

## Introduction

Over the past 25 y, and in parallel with rapid economic growth, Vietnam has introduced a system of compulsory health insurance for poor and vulnerable groups. Eligibility has progressively expanded to populations deemed in need of coverage.^1^ In addition, a system of voluntary health insurance has been introduced for those not covered by the compulsory scheme. By 2022, an estimated 87% of the population were covered by either compulsory or voluntary health insurance.^2^ Alongside insurance roll-out, the Vietnam Health Financing Strategy 2016–2025 set out to reduce out of pocket (OOP) expenditure for healthcare to < 40% of total healthcare expenditure by 2020, and to < 30% by 2025^3^; although there has been progress towards this goal, OOP expenditure still accounted for 40% of total healthcare expenditure in 2021.^4^ One potential reason for this is that the schedule of reimbursement set out by the Vietnam Social Security System has not increased in line with rapid inflation for goods and services in the country, and the strong decentralisation of health financing for hospitals means that they need to charge patients a size-able copayment to make up this difference.^5^ Households can experience financial hardship when the burden of OOP payments for healthcare exceeds their capacity to pay; such expenditure may mean that people have to cut down on essentials, such as food, clothing and payments for education.^6^ Although definitions vary, OOP payments are considered catastrophic health expenditure (CHE) when the sum exceeds a predefined threshold (generally 10% or 25%) of a household’s total income.^7^

Previous studies have reported the direct medical costs for patients with dengue shock (DS) and septic shock (SS) in Vietnam; the key findings were that CHE was common, and that critical care is very expensive relative to ward-level care.^8, 9^ However, these studies did not account for the prehospital and posthospital healthcare costs, the direct non-medical or productivity costs of the wider episode of critical illness.^10^ Therefore, we conducted a prospective observational study, aiming to report the direct medical and non-medical costs associated with DS and SS, including prehospital and posthospital direct medical costs and productivity costs for patients and their carers until 6 mo postdischarge. We also sought to estimate the proportion of patients facing CHE and catastrophic costs, report sources of money to pay for the episode and the proportion of patients pushed into debt due to the cost of illness. The aim of this study was to estimate the true cost of these illnesses for the patient and their household.

## Methods

### Study procedures

We conducted a prospective observational study at the Hospital for Tropical Diseases (HTD), a tertiary referral centre for infectious diseases in Ho Chi Minh City, Vietnam, from 2019 to 2021. Ethical approval was obtained from the Oxford Tropical Research Ethics Review Committee and the Ethics review committee at HTD. Written informed consent was obtained from all participants. We recruited patients aged ≥16 y within 24 h of diagnosis of DS or SS. Diagnostic criteria for DS and SS were in line with the 2009 WHO and Sepsis 3 definitions, respectively.^11, 12^ We excluded patients who were pregnant, patients with DS admitted after day 7 of illness and patients who developed SS >48 h after admission to hospital.

Hospital bills were collected, and an economic questionnaire was conducted at hospital discharge, 1, 3 and 6 mo later. Briefly, the questionnaire included questions on direct medical costs prior to hospital admission and after discharge, non-medical costs including accommodation, transport, food and productivity costs for the patient and their carers. In cases where the patient did not survive, only data on the hospital bill, insurance status and percentage copay were collected from hospital records; no economic questionnaires were conducted. Preliminary results were presented at the International Congress on Infectious Diseases, Kuala Lumpur, 2022.^13^

### Statistical analysis

Statistical analysis was performed in Stata version 17.0 (Stata-Corp, TX, USA). Binary endpoints are summarised as n (%), and because cost data were non-parametric and skewed, endpoints are reported as median (25th; 75th centiles).

The cost data were adjusted for inflation to 2020 values in Vietnamese Dong using the World Bank gross domestic product deflators.^14^ Inflation-adjusted costs are reported in US$; the conversion was made using the 2020 average local currency unit to the US$ rate.^15^

### Direct medical and direct non-medical costs

Direct medical costs were calculated from both the hospital bill obtained at the end of the inpatient stay, and patient-reported OOP costs for healthcare visits preadmission and postadmission. Direct non-medical costs were calculated as a total of transport, accommodation and food costs estimated by the patient/carer during the economic questionnaire.

### Productivity costs

Patient productivity costs were estimated using the human capital approach.^10^ For patients in paid work, a daily wage was calculated by dividing their self-reported individual monthly net income by 24 (based on a standard 6-d working week).^16^ For patients who would otherwise have been undertaking unpaid work, or in education, the minimum wage was applied to value their time. Productivity costs associated with presenteeism were not quantified in this analysis, nor were productivity costs associated with fatal cases. The time spent by carers either in attendance during the hospital admission, assisting the patient on posthos-pitalisation healthcare visits, or providing informal care at home postdischarge, was calculated using the replacement/proxy cost approach^17^; carer time was valued at the minimum wage on the basis that another individual employed for caring duties would likely be employed at this rate.

The minimum wage was calculated as follows: the Vietnamese government assigns each district one of four categories with varying minimum wages depending on the perceived cost of living within each.^18^ The address for each enrolled patient was crosschecked against this list, and a system of weighted averages was used to calculate an average minimum wage for the patient population (using 2020 values). The average minimum wage applied to the population in this study was US$169.53 per month, or US$7.06 per day.

Patient productivity costs were split between (i) prehospital (time spent attending healthcare visits prior to admission); (ii) in hospital; and (iii) postdischarge (including both time off work following discharge and time spent attending further healthcare visits after the patient had returned to work). Healthcare visits that lasted <4 h were valued at half a day, and visits >4 h were counted at face value. Productivity costs were calculated for up to 6 mo after discharge. The patient productivity cost was calculated as the number of days/part days multiplied by either (i) the daily wage if the patient was in prior paid work, or (ii) the daily minimum wage if the patient was performing unpaid work or in education prior to their illness.

The carer productivity loss was calculated for the hospital admission and the period up to 6 mo postdischarge; data were not collected for the prehospital phase. We calculated the lost productivity for each day the carer spent at the hospital by applying the minimum wage for each working day. Carer productivity loss postdischarge was calculated as the sum of (i) the time carers spent accompanying patients on postdischarge healthcare visits/in attendance during readmissions to hospital; and (ii) the time they spent providing informal care to the patient at home.

### Catastrophic health expenditure and catastrophic costs

CHE was estimated using the budget share approach, with OOP payments for healthcare as the numerator and patient-reported total household income as the denominator.^7^ CHE was defined as OOP direct medical costs amounting to either >10% or >25% of the total annual (preillness) household income; both thresholds are specified as indicators of progress toward Sustainable Development Goal 3.8 (indicator 3.8.2).^19, 20^ An estimate of the proportion facing catastrophic costs is also reported; this was defined as both direct costs and productivity losses associated with the illness episode exceeding 20% of the preillness household annual income, in line with recommendations from the WHO and extensive literature from the TB field.^21, 22^

## Results

We recruited 122 patients with DS, of whom 118 survived, and 35 patients with SS, of whom 24 survived to hospital discharge. Table 1 summarises the demographics, insurance status, employment and income for the patient and their household. The majority of patients in both groups were engaged in either self-employment, paid work, education or unpaid working roles (e.g. subsistence farming, looking after a family). For those in earning roles, the median (25th; 75th centiles) monthly income was US$224 (86; 388) for DS and US$215 (129; 302) for SS. The median number of adults in the household was three in both the DS and SS groups, with median household income US$646 (431; 4309) and US$517 (345; 646) for DS and SS, respectively. A greater proportion of patients with SS had health insurance (68.6% for SS vs 57.4% for DS) and the majority of those insured had a 20% copayment (90.0% for DS and 58.3% for SS). No patients with SS and six patients with DS had private health insurance via a non-governmental scheme.

### Preadmission costs

Table 2 provides a breakdown of direct medical, non-medical and indirect costs incurred preadmission. The majority of patients (91.4% with DS and 62.5% with SS) had sought healthcare for their illness prior to HTD admission; most of these had been an inpatient at another hospital (Supplementary Table 1). OOP costs for these healthcare encounters were reported as median (25th; 75th centiles) US$30 (14; 65) for DS and US$25 (0; 60) for SS (Table 2). For most, direct non-medical costs including the cost of food and accommodation during the healthcare episodes prior to HTD admission were negligible.

Patients with DS lived a median 24.5 (9; 86) km, and those with SS lived 22 (11; 73) km, from the hospital. Most patients with DS arrived by ambulance, but the cost of ambulance hire was reimbursable by insurance for only seven patients (10% of those travelling by this method). Fewer patients with SS arrived by ambulance (25%), none of whom were eligible for cost reimbursement. The median (25th; 75th centiles) cost of travel from home to hospital was US$22 (3; 52) for DS and US$6 (0; 43) for SS. Most patients in both groups elected to travel home using a cheaper method of transport (Supplementary Table 1).

### Costs during hospital admission

Table 2 summarises the hospital bills and OOP expenses (total hospital bill minus insurance contribution, if insured) paid by patients. The distribution of costs is markedly skewed, with a few patients in each illness group having very high OOP expenses (Supplementary Figures 1 and 2). Additional medical/medication costs incurred during the hospital admission, but not included on the hospital bill, were reported by 48% and 83% of patients with DS and SS, respectively; these were median (25th; 75th centiles) US$9 (6; 22) for DS and US$47 (22; 82) for SS.

Considering the OOP expenses arising from the hospital bill only, 18.9% and 71.4% patients with DS and SS, respectively, incurred CHE, with the threshold set at > 10% of annual household income. When the threshold was moved to >25% of annual household income, the proportions incurring CHE were 15.8% for DS and 60.0% for SS. Having health insurance offered slight protection against CHE for patients with DS, but paradoxically, the proportion of patients with CHE was higher in the insured vs uninsured with SS. Despite having government health insurance, 62.5% of patients with SS incurred CHE at > 25% of annual household income.

Most patients received care from a relative/friend during their admission; most had two informal caregivers for at least part of the hospital stay (Supplementary Table 2). The median (25th; 75th centiles) total number of hours that both carers spent at the hospital (day or night) was 144 (104; 192) for DS and 384 (312; 868) for SS. Most carers were first-degree relatives or spouses, who would otherwise have been in formal paid work, self-employment or performing unpaid work (looking after family, subsistence farming). The cost of food per day during the hospital admission for patients and their carers was estimated at median (25th; 75th centiles) US$9 (6; 11). Transport and accommodation costs for carers were negligible (Table 2).

### Costs after hospital discharge

Opposite patterns were seen for healthcare utilisation between hospital discharge and 6-mo follow-up. After discharge, patients with DS had few further contacts with healthcare providers, and among those who did, the majority (75% at 1 mo, 87% at 3 mo, 91% at 6 mo) attributed these encounters to a new medical concern, rather than related to DS (Supplementary Table 3). No patient with DS was readmitted to hospital within the first month after discharge. The median OOP costs for those requiring health-care visits was US$9 at months 1 and 3, and US$22 at month 6 postdischarge (Table 2). Most patients recovering from DS did not need assistance to attend subsequent healthcare visits, or for activities of daily living after discharge (Supplementary Table 3). Among those in paid work prior to their illness, patients with DS had largely returned to work by the 1-mo follow-up visit (median days off work postdischarge=7) (Table 3).

By contrast, patients recovering from SS reported frequent healthcare encounters after discharge, and the majority (75% at 1 mo, 88% at 3 mo, 80% at 6 mo) attributed these health-care visits to continued problems from their episode of SS (Supplementary Table 3). Also, 37.5% reported an inpatient admission to either a government or private hospital in the first month after discharge. The median OOP cost for additional healthcare visits was US$142 at 1 mo, US$23 at 3 mo and US$97 at 6 mo postdischarge (Table 2). Patients with SS also reported subsistence costs for these healthcare visits. Most patients (79%) required a caregiver to help to travel to appointments and assist with activities of daily living during the first month after discharge (Supplementary Table 3). Among those in paid work prior to their illness, most patients with SS had a delayed return to work (median days off work postdischarge=84), if they returned at all by 6 mo follow-up (Table 3).

### Productivity costs and catastrophic costs

Table 2 summarises the estimated productivity costs for both patients and their carers until 6 mo postdischarge. Patients with DS accrued a median (25th; 75th centiles) US$123 (92; 252) in productivity costs; most of these occurred during the hospital admission and immediately postdischarge. By contrast, patients with SS accrued median (25th; 75th centiles) US$780 (629; 1043) in productivity costs; most of the lost earnings occurred during the postdischarge period.

Productivity losses for carers were not captured during the pre-hospital phase. From the time of admission until 6 mo posthospital discharge, estimated median (25th; 75th centiles) carer productivity losses for patients with DS were US$42 (28; 56) and US$327 (90; 789) for patients with SS.

Table 2 provides an estimate of the total cost of the illness for the patient (and their household), including OOP direct medical and non-medical costs (prehospital, admission and first month postdischarge) plus productivity costs for patients and their carers until 6 mo postdischarge. The median (25th; 75th centiles) total cost was US$407 (309; 560) for DS and US$2404 (1702; 3025) for SS. Fourteen patients with DS (12%) and 17 with SS (74%) incurred catastrophic costs, with the threshold set at > 20% of annual household income.

### Sources of money and debt

Table 3 summarises the sources from which the patients and their families obtained the money to pay for expenses related to the hospital admission. Most either took the money from their own savings (60% for DS, 42% for SS) or borrowed from relatives (36% for DS, 46% for SS). No patient borrowed from a formal credit source.

The cost of illness led 44 patients with DS (38%) and 15 with SS (52%) to have new debt at the time of discharge from hospital. The median (25th; 75th centiles) debt was US$431 (215; 625) for DS and US$862 (646; 1724) for SS. Fewer patients in each group had persistent debt at each subsequent follow-up appointment, but the median amount of debt for those who had not cleared it either stayed stable in the case of DS, or increased in the case of SS (Table 3).

## Discussion

We have presented the direct medical, non-medical and productivity costs incurred during an episode of DS or SS in Vietnam, providing a window into the ‘true’ cost for patients surviving critical infectious diseases.

The most striking finding is the marked increase in cost of illness once prehospital and posthospital direct medical, non-medical and productivity costs have been accounted for. The median total cost for patients with DS was US$407, of which US$165 (41%) was attributable to patient and carer productivity costs. For those with SS, the median total cost was US$2404, of which US$1107 (46%) were productivity costs. For both DS and SS, the total cost of illness was 6–7 times greater than the OOP contribution to the inpatient hospital bill alone, suggesting that the latter is an insensitive measure of the economic shock associated with critical illness due to acute infection.

The relatively larger cost of SS in this study was attributable to a combination of increased cost of medical care during admission, increased ongoing need for healthcare following discharge and marked productivity losses from both patients and carers for a sustained period postdischarge. The relative burden of productivity costs in SS was much higher compared with DS for several reasons: patients with SS were older with more comorbidities at baseline and had higher severity of illness (measured by organ failure); this contributed to longer ICU and hospital stays. At the study site, the carers (often more than one) needed to be at the hospital 24 h per day if their relative was admitted to the ICU. Patients with SS were off work for longer after discharge, needed rehospitalisation and follow-up appointments more frequently and were more often discharged with an ongoing need for informal care at home. We found that the majority of carers would otherwise have been in paid work, and our choice to measure their productivity costs using the more conservative cost replacement method may have underestimated the true loss of income to the household.^10^ One-half of the patients with SS did not return to work at all by 6 mo; while no studies could be found evaluating return to work following SS in lower-middle income countries, prolonged inability to work is well described following ICU admission with sepsis in high-income settings.^23 , 24^ Although CHE is a widely used indicator of performance of national health insurance coverage plans, our analysis indicates that not considering the wider cost of critical illness greatly underestimates the burden placed on households by non-healthcare costs. Especially in the case of SS, critical illness has chronic consequences and, as such, we suggest that reporting catastrophic costs should be more commonplace.^13^

The results from this study replicate our previous finding that having insurance conferred only minor protection against CHE for OOP costs during hospitalisation for DS.^9^ These findings echo those from two population-level studies in Vietnam, which reported that health insurance membership did not significantly reduce the likelihood of a household experiencing CHE, or provide protection against impoverishment due to OOP payments for healthcare.^24, 26^ To meet universal health coverage goals,^27^ the next phase of health insurance roll-out in Vietnam will need to address how to extend financial protection to the small minority requiring high-cost care such as ICU admission and organ support interventions. This may involve commitment by the government to cover the shortfall between the true cost of providing critical care and the reimbursement schedules, or the introduction of a supplementary critical illness insurance package. The latter was adopted by China in 2012, but some analyses have shown that the programme has had minimal impact on reducing the incidence of CHE, and has even increased medical expenditure among beneficiaries, in part because patients accessed treatment more often, and at higher level hospitals.^28–30^ However, Thailand has had great success in implementing a universal healthcare strategy with comprehensive cover including high-cost interventions, and no copayment at the point of care.^31^ Although the budgetary commitment required to achieve this may be currently out of reach for Vietnam, Thailand has shown that it is possible with sustained political will.^32^ In addition, the rapidly developing critical care sector in lower-middle income countries may provide opportunities to reimagine how to deliver intensive care at a lower cost, by utilising novel technologies and digital health approaches.^33^

Paradoxically, we found that the proportion of patients with SS and CHE was higher among the insured vs uninsured (75% vs 64%, respectively). Although this finding may be a function of small patient numbers, it may also reflect differential decision making. Qualitative studies conducted with both healthcare professionals and families of critically ill patients in lower-middle income countries indicate that the cost of care is a key factor influencing decision making in an ICU setting.^34, 35^ In our study, families may have opted out of high-cost interventions such as haemofiltration when the patient did not have insurance. Although literature on the perception and burden of financial stress in the context of critical illness in lower-middle income countries is sparse, qualitative research from the USA suggests that the burden of financial stress is high both during the acute illness and for at least 6 mo thereafter, and that families would value financial guidance from their healthcare providers at several points during and following hospitalisation.^36, 37^ However, different models of healthcare financing and social protection limit geographical extrapolation of these findings. Going forward, a combination of quantitative and qualitative research methods will be required to understand the influence of insurance status and family financial strain on decision making in the Vietnamese context.

Most patients managed their healthcare costs through either their own savings or by borrowing from informal sources; we did not ask whether informal credit was preferred, or whether this route was chosen because formal credit was out of reach. The median amount borrowed was typically much higher than the hospital bills, which reinforces the key finding of this study: that many of the costs associated with critical illness are not represented in the hospital bill. Formalising a central fund or community financing initiative from which households experiencing catastrophic costs in the aftermath of critical illness can borrow at low/no interest may provide protection against the full economic shock of critical illness during the acute phase.

Our study has several limitations. First, we did not include estimates of CHE or catastrophic costs for families of patients who did not survive to hospital discharge. Sensitively designed, qualitative research will need to address this knowledge gap, with a view to advocating for a financial protection system that functions regardless of survival outcome. Second, we did not ask questions to understand the impact of insurance status or family financial strain on clinical decision making. Third, we did not estimate the cost of presenteeism, either before or after hospital admission with shock. Building these estimates, plus qualitative methodology into future study designs, would help to improve the accuracy and interpretation of our findings.

## Conclusions

We have presented a detailed, patient-orientated analysis of the cost of DS and SS in Vietnam. The study highlights that the true cost of these critical illnesses is several times greater than the hospital bill, and that the burden of OOP costs and productivity losses is borne almost entirely by borrowing from savings and informal sources. Further, healthcare insurance did not offer substantial protection against CHE. As critical care capabilities expand in Vietnam, it is urgent to consider central provision to protect the small number of patients requiring high-cost intensive care from financial catastrophe or impoverishment.

## Supplementary Material

Supplementary data are available at International Health online.

Supplementary file

## Figures and Tables

**Table 1:** Patient demographics, income and insurance status

Variable	n	Dengue shock	n	Septic shock
Demographics				
Age, yˆ	118	26 (20; 33)	37	55 (47; 63)
Male sex*		58 (49)		23 (62)
Marital status*	118		24	
Married		53 (44.9)		18 (75.0)
Single		59 (50.0)		3 (12.5)
Divorced/separated/widowed		6 (5.1)		3 (12.5)
Preillness employment status*	118		24	
Full-time paid work		63 (53.4)		10 (41.7)
Part-time paid work		7 (5.9)		0 (0)
Looking after home/family		1 (0.9)		5 (20.8)
Full-time education		24 (20.3)		0 (0)
Subsistence farming		7 (5.9)		2 (8.3)
Self-employed		14 (11.9)		4 (16.7)
Unemployed/retired		2 (1.7)		3 (12.5)
Patient monthly net incomeˆ	118	224 (86; 388)	24	215 (129; 302)
Household dataˆ				
Number of people in household		4 (3; 5)		4 (4; 5)
Adults		3 (2; 4)		3 (3; 4)
Children		1 (0; 2)		1 (1; 2)
Monthly household income		646 (431; 4309)		517 (345; 646)
Health insurance*	122		35	
Any health insurance policy		70 (57.4)		24 (68.6)
Government insurance only		64 (95.6)		24 (100)
Government and private insurance		6 (9.0)		0 (0)
Copayment percentage				
No copayment		7 (10.0)		9 (37.5)
5%		0 (0)		1 (4.2)
20%		63 (90.0)		14 (58.3)

Incomes are in US$, rounded to the nearest dollar.

* n (%).

ˆ median (25th; 75th centiles).

**Table 2:** Costs associated with episodes of dengue shock and septic shock

Variable	n	Dengue shock	n	Septic shock
**PREADMISSION COSTS**				
Direct medical cost				
Incurred healthcare costs prior to HTD admission*	116	106 (91.4)	24	15 (62.5)
Total cost of prior healthcareˆ	106	35 (11; 56)	15	26 (0; 60)
OOP costˆ	106	30 (14; 65)	15	25 (0; 60)
Direct non-medical costs				
Cost of food, accommodation during prior healthcareˆ	106	0 (0; 7)	15	0 (0; 2)
Cost of travel to HTDˆ	112	22 (3; 52)	24	6 (0; 43)
Indirect costs				
Patient productivity loss during prior healthcareˆ	116	3 (1; 14)	15	0 (0; 2)
**ADMISSION COSTS**				
Direct medical cost				
Total hospital billˆ	120	94 (77; 140)	34	1407 (888; 3803)
Hospital bill per day of admissionˆ	120	18 (15; 25)	34	114 (73; 380)
OOP contribution to hospital billˆ		64 (19; 99)		357 (258; 1245)
Reported medical costs additional to hospital bill*	118	59 (48.8)	24	20 (83.3)
Additional costsˆ		9 (6; 22)		47 (22; 82)
Direct non-medical costs				
Food cost per day for patient and carerˆ	112	9 (6; 11)	24	7 (4; 9)
Cost of travel from hospital to homeˆ		9 (3; 14)		4.3 (0; 35)
Travel and accommodation cost for carerˆ		0 (0; 0)		0 (0; 0)
Indirect costs				
Patient productivity lossˆ	117	58 (42; 97)	24	86 (50; 297)
Carer productivity lossˆ		42 (28; 56)		106 (71; 177)
**POSTDISCHARGE COSTS (6 mo)**				
Direct medical costs				
OOP cost incurred for healthcare postdischarge:				
Discharge: 1-mo follow-upˆ	18	9 (5; 345)	18	142 (108; 345)
1–3 mo follow-upˆ	15	9 (2; 22)	15	23 (8; 78)
3–6 mo follow-upˆ	11	22 (13; 43)	10	97 (14; 194)
Direct non-medical costs				
Travel, food, accommodation cost incurred for healthcare:				
Discharge: 1-mo follow-upˆ	18	0 (0; 0)	18	52 (22; 86)
1–3-mo follow-upˆ	15	0 (0; 0)	15	172 (129; 215)
3–6-mo follow-upˆ	11	0 (0; 0)	10	43 (22; 215)
Indirect costs				
Patient productivity lossˆ	117	50 (50; 119)	24	595 (431; 905)
Carer productivity lossˆ		0 (0; 0)		131 (0; 440)
**CHE**				
CHE (10% threshold) for OOP healthcare costs (admission only)*	120	24 (18.9)	34	25 (71.4)
CHE (10%) with no health insurance*		10 (19.2)		7 (63.6)
CHE (10%) with health insurance*		9 (12.9)		18 (75.0)
CHE (25% threshold) for OOP healthcare costs (admission only)*		20 (15.8)		21 (60.0)
CHE (25%) with no health insurance*		7 (13.5)		6 (54.6)
CHE (25%) with health insurance*		8 (11.4)		15 (62.5)
**CATASTROPHIC COSTS**				
Estimated cost of illness for the patient: OOP direct medical and non-medical costs	117	407 (309; 560)	23	2404 (1702; 3025)
(prehospital, admission, 1-mo postdischarge) + productivity costs for patient and carers to 6-mo postdischargeˆ				
Catastrophic costs (20% threshold)*		14 (12.0)		17 (73.9)

CHE: catastrophic health expenditure; HTD: Hospital for Tropical Diseases; OOP: out of pocket.

All costs are in US$, rounded to the nearest dollar (cost year 2020).

* n (%).

ˆ median (25th; 75th centiles).

**Table 3:** Sources of money, debt and return to work

Variable	n	Dengue shock	n	Septic shock
Return to work after hospital discharge*	117		24	
In employment prior to illness		87 (74.4)		16 (66.7)
If yes, returned to work by:				
1-mo follow-up visit		80 (92.0)		3 (18.8)
3-mo follow-up visit		83 (95.4)		5 (31.3)
6-mo follow-up visit		82 (94.3)		8 (50.0)
Median days off work postdischarge		7		84
Sources of money to pay for illness episode*	117		24	
Own savings		70 (59.8)		10 (41.7)
Borrowed from relatives		42 (35.9)		11 (45.8)
Borrowed from black market		1 (0.9)		1 (4.2)
Borrowed from bank		0 (0)		0 (0)
Sale of personal property		1 (0.9)		0 (0)
Other (gift, salary advance, charity)		3 (2.6)		2 (8.3)
If cost of illness led to debt:	117		24	
Debt at hospital discharge*		44 (37.6)		15 (51.7)
If yes, amount of debtˆ		431 (215; 625)		862 (646; 1724)
Persistent debt at 1 mo*		31 (70.5)		14 (93.3)
If yes, amount of debtˆ		431 (215; 732)		905 (776; 1724)
Persistent debt at 3 mo*		18 (42.9)		10 (90.9)
If yes, amount of debtˆ		409 (215;560)		948 (646; 2154)
Persistent debt at 6 mo*		5 (13.2)		6 (66.7)
If yes, amount of debtˆ		409 (302; 431)		1508 (862; 2585)

All costs are in US$, rounded to the nearest dollar (cost year 2020).

* n (%).

ˆ median (25th; 75th centiles).

## Data Availability

The underlying data can be accessed in the Oxford University Research Archive by the following 10.5287/ora-eoqj4zg89.
