# Supplementary material for: The cost of dengue shock and septic shock in Vietnam: a patient-centred economic analysis
Source: Int Health. 2025 Sep 23;18(3):431–9. doi: 10.1093/inthealth/ihaf105 (PMC7618728; doi:10.1093/inthealth/ihaf105)

Supplementary figure 1: Box plot of direct medical cost for hospital admission in USD for patients with dengue shock and septic shock.


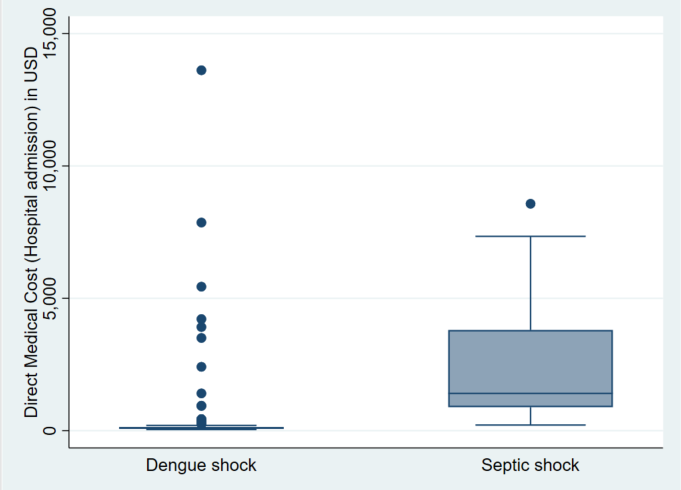


Supplementary figure 2: Box plot of direct medical cost per day of hospital admission in USD for patients with dengue shock and septic shock


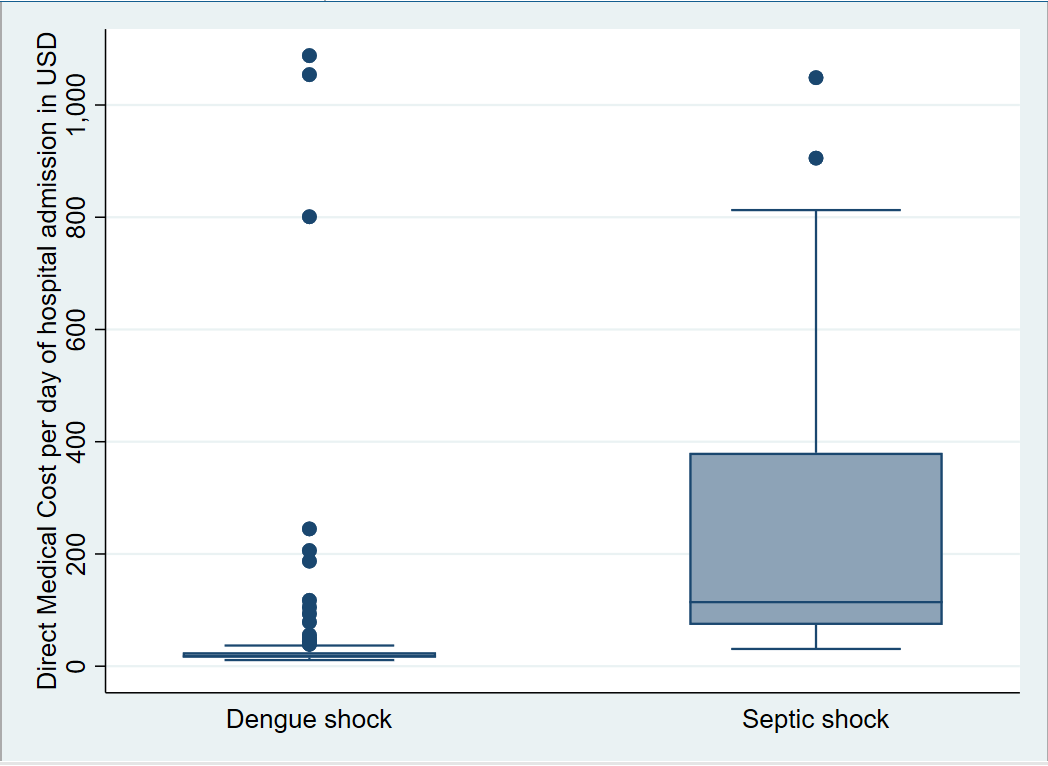

Supplement: ihaf105_Supplemental_Files [file ihaf105_supplemental_files.zip › Supplementary figure 1 and 2.docx]
