# Supplementary material for: The cost of dengue shock and septic shock in Vietnam: a patient-centred economic analysis
Source: Int Health. 2025 Sep 23;18(3):431–9. doi: 10.1093/inthealth/ihaf105 (PMC7618728; doi:10.1093/inthealth/ihaf105)
Supplement: ihaf105_Supplemental_Files [file ihaf105_supplemental_files.zip › Supplementary tables 1-3.docx]

Supplementary Table 1: Pre-admission direct medical and non-medical costs

| **Variable** | **n** | **Dengue shock** | **n** | **Septic shock** |
| --- | --- | --- | --- | --- |
| **Sought healthcare for this illness prior to HTD admission*** | 116 | 106 (91.4) | 24 | 15 (62.5) |
| Type of healthcare received*  Visited a pharmacy  Private outpatient clinic  Government outpatient clinic  Inpatient at another hospital |  | 1 (0.9)  15 (13.5)  11 (9.9)  83 (74.8) |  | 1 (6.7)  1 (6.7)  3 (20.0)  10 (66.7) |
| Total bill for prior healthcare^  OOP cost for prior healthcare^  Time taken for prior healthcare (hours)^  Cost of food, accommodation^ |  | 35 (11; 56)  30 (14; 65)  20 (4; 45)  0 (0; 6.5) |  | 25 (0; 60)  25.9 (0; 60)  24 (4; 48)  0 (0; 2.2) |
| **Cost of transport to and from hospital** | 112 |  | 24 |  |
| Distance from home to hospital (km)^ |  | 24.5 (9; 85.5) |  | 22 (11; 73) |
| Time taken from home to hospital (mins)^ |  | 60 (30; 120) |  | 60 (25; 105) |
| Method of transport*  Ambulance  Ambulance bill covered by health insurance  Own motorbike  Own car  Taxi  Hired motorbike (e.g. Grab app)  Other |  | 71 (60.2)  7 (9.9)  21 (17.8)  10 (8.5)  14 (11.9)  1 (0.9)  1 (0.9) |  | 6 (25.0)  0 (0)  5 (20.8)  5 (20.8)  6 (25.0)  0 (0)  2 (8.3) |
| Cost of travel from home to hospital^ |  | 22 (3; 52) |  | 6 (0; 43) |
| Returned home by same transport* |  | 31 (26.3) |  | 7 (29.2) |
| Cost of travel from hospital to home^ |  | 9 (3; 14) |  | 4.3 (0; 35) |

* n (%), ^ Median (25^th^; 75^th^ centiles) [range], OOP: out of pocket, all costs are in USD, rounded to nearest dollar (cost year 2020).

Supplementary table 2: role of informal carers during hospital admission, relationship to patient and time spent at hospital

| **Variable** | **n** | **Dengue shock** | **n** | **Septic shock** |
| --- | --- | --- | --- | --- |
| **Informal care by relatives/friends during admission**  Received care from relative/friend for more than 4 hours*  Total hours informal carers spent at hospital^  Number of informal caregivers*  1  2 | 118 | 118 (100)  144 (104; 192)  47 (39.83)  71 (60.17) | 24 | 23 (95.83)  384 (312; 868)  10 (41.67)  14 (58.33) |
| **Primary informal caregiver**  Relationship to patient*  Mother  Father  Partner/spouse  Sibling  Other relative  Friend  Employment status*  Full time paid work  Part time paid work  Looking after home/family  Student/pupil  Subsistence farming (unpaid)  Self employed  Unemployed  Retired  Time spent caring at hospital in hours (day & night)^ | 118 | 52 (44.07)  20 (16.95)  27 (22.88)  14 (11.86)  4 (3.39)  1 (0.85)  47 (39.83)  1 (0.85)  19 (16.10)  1 (0.85)  21 (17.8)  22 (18.64)  3 (2.54)  4 (3.39)  114 (72; 144) | 24 | 4 (16.67)  0 (0)  10 (41.67)  1 (4.17)  9 (37.5)  0 (0)  14 (58.33)  1 (4.17)  2 (8.33)  0 (0)  3 (12.50)  4 (16.67)  0 (0)  0 (0)  240 (168; 396) |
| **Secondary informal caregiver**  Relationship to patient*  Mother  Father  Partner/spouse  Sibling  Other relative  Friend  Employment status*  Full time paid work  Part time paid work  Looking after home/family  Student/pupil  Subsistence farming (unpaid)  Self employed  Unemployed  Retired  Time spent caring at hospital in hours (day & night)^ | 71 | 14 (19.72)  20 (28.17)  8 (11.27)  14 (19.72)  12 (16.90)  3 (4.23)  32 (45.07)  3 (4.23)  9 (12.68)  2 (2.82)  10 (14.08)  11 (15.49)  3 (4.23)  1 (1.41)  48 (16; 96) | 14 | 1 (7.14)  0 (0)  0 (0)  1 (7.14)  10 (71.43)  2 (14.29)  6 (42.86)  0 (0)  3 (21.43)  1 (7.14)  1 (7.14)  3 (21.43)  0 (0)  0 (0)  120 (40; 132) |

* n (%), ^ Median (25^th^; 75^th^ centiles)

Supplementary Table 3: Post-discharge healthcare usage and informal care needs

| **Variable** | **n** | **Dengue shock** | **n** | **Septic shock** |
| --- | --- | --- | --- | --- |
| **Costs between discharge & 1 month follow up**  **Healthcare usage**  Required healthcare between discharge & 1 month*  Type of healthcare received*  Pharmacy  Traditional medicine  Private outpatient clinic  Government outpatient clinic  Inpatient (private or government)  Reason for seeking healthcare*  Continuing problems from original illness  New illness  Needed carer to accompany on healthcare visits*  Carer time spent on healthcare visits (hours)^ | 117 | 18 (15.4)  n=20 visits  7 (35.0)  0 (0)  5 (25.0)  8 (40.0)  0 (0)  5 (25.0)  15 (75.0)  9 (45.0)  5 (3; 24) | 24 | 18 (75.0)  n=24 visits  0 (0)  1 (4.2)  0 (0)  14 (58.3)  9 (37.5)  18 (75.0)  6 (25.0)  19 (65.6)  4 (3; 168) |
| **Informal care needs**  Needed informal care at home*  Hours requiring informal care^  Days requiring informal care^  Time requiring informal care per day in hours^  Main activity of carer (if not providing informal care)*  Paid work  Household chores, caring for children or relatives  Attending school or university  Subsistence farming  Self employed | 117  11^$^ | 10 (8.6)  60 (30; 84)  7 (3; 10)  12 (6; 12)    4 (40.0)  3 (30.0)  1 (10.0)  1 (10.0)  2 (20.0) | 24  25^$^ | 19 (79.2)  216 (112; 248)  28 (14; 30)  8 (4; 12)  10 (40.0)  3 (12.0)  0 (0)  2 (8.0)  10 (40.0) |
| **Costs between 1 & 3 month follow-up**  **Healthcare usage**  Required healthcare between 1 & 3 month follow-up*  Type of healthcare received*  Pharmacy  Traditional medicine  Private outpatient clinic  Government outpatient clinic  Inpatient (private or government)  Reason for seeking healthcare*  Continuing problems from original illness  New illness  Needed carer to accompany on healthcare visits*  Carer time spent on healthcare visits in hours^ | 113  15  15  15 | 15 (13.3)  6 (42.9)  2 (14.3)  2 (14.3)  3 (21.4)  1 (7.1)  2 (13.3)  13 (86.7)  2 (13.3)  2.5 (1; 4) | 22  15  15  15 | 15 (68.2)  4 (26.7)  0 (0)  1 (6.7)  7 (46.7)  3 (20.0)  13 (87.7)  2 (13.3)  9 (60.0)  5 (1; 480) |
| **Informal care needs**  Needed informal care at home between 1-3 months*  Hours requiring informal care^ | 113 | 2 (1.8)  2.5 (1; 4) | 22 | 8 (36.4)  244 (142; 540) |
| **Costs between 3 & 6 month follow-up**  **Healthcare usage**  Required healthcare between 3 & 6 month follow up*  Type of healthcare received*  Pharmacy  Traditional medicine  Private outpatient clinic  Government outpatient clinic  Inpatient (private or government)  Reason for seeking healthcare*  Continuing problems from original illness  New illness  Needed carer to accompany on healthcare visits*  Carer time spent on healthcare visits in hours^ | 113  11  11  11 | 11 (9.7)  4 (36.4)  0 (0)  4 (36.4)  2 (18.2)  1 (9.1)  1 (9.1)  10 (90.9)  4 (36.4)  4 (2.5; 4.5) | 19  10  10  10 | 10 (52.6)  1 (10.0)  1 (10.0)  0 (0)  4 (40.0)  4 (40.0)  8 (80.0)  2 (20.0)  7 (70.0)  72 (4; 456) |

* n (%), ^ Median (25^th^; 75^th^ centiles) [range], OOP: out of pocket, all costs are in USD, rounded to nearest (cost year 2020). Carer productivity costs were not calculated for pre-hospital healthcare visits. ^$^ Some patients had more than one informal carer
